# The preclinical efficacy of the novel hypomethylating agent NTX-301 as a monotherapy and in combination with venetoclax in acute myeloid leukemia

**DOI:** 10.1038/s41408-022-00664-y

**Published:** 2022-04-11

**Authors:** Byungho Lim, Dabin Yoo, Younghwa Chun, Areum Go, Kyung-Jin Cho, Daeun Choi, Myoung Eun Jung, Ha Young Lee, Rebecca J. Boohaker, Jin Soo Lee, DooYoung Jung, Gildon Choi

**Affiliations:** 1 Research Center for Drug Discovery Technology, Korea Research Institute of Chemical Technology, Daejeon, Republic of Korea; 2 Pinotbio, Inc, Suwon, Republic of Korea; 3 Southern Research, Division of Drug Discovery, Birmingham, AL USA

**Keywords:** Acute myeloid leukaemia, Drug development

**Dear Editor**,

Intensive induction chemotherapy, the standard of care for patients with acute myeloid leukemia (AML), is sufficiently effective to achieve complete remission in 60–85% of younger patients (<60 years) [1, 2]. However, the use of intensive regimens is often limited in elderly patients (≥70 years), who frequently have comorbidities and poor performance status. Given that the median age at AML diagnosis is 67 years and that approximately one-third of patients are >75 years [3], many patients are not suitable for intensive chemotherapy. Thus, their clinical management remains challenging.

As an alternative strategy, less-intensive hypomethylating agents (HMAs) [e.g., azacitidine (AZA) and decitabine (DAC)] alone or in combination with the BCL-2 inhibitor venetoclax (VCX) [4] are currently being used in these unfit patients [5]. Compared with patients treated with standard chemotherapy, patients administered HMA monotherapy achieved lower complete remission rates of 20–30% [2], although HMAs conferred modest overall survival benefits [5]. In contrast, patients treated with the AZA + VCX combination achieved complete response rates of ~60–70% and exhibited a median overall survival time that was ~1.5-fold longer than that of patients treated with AZA alone [6, 7]. Despite this major breakthrough, the low response rates to conventional HMAs when administered alone and the adverse events with grade ≥3 that occur when HMAs are administered in combination with VCX highlight the need for further improvement of these regimens [8].

Here, we report a novel HMA, 5-aza-4′-thio-2′-deoxycytidine (NTX-301), and emphasize its improved therapeutic index. Based on six animal studies and transcriptome analyses, we aim to thoroughly investigate the preclinical efficacy of NTX-301 as a monotherapy and in combination with VCX by performing comparative analyses with the conventional agents AZA and DAC.

We evaluated the preclinical efficacy of NTX-301 monotherapy by establishing four different mouse models encompassing both systemic and subcutaneous xenografts. The animal care and use program for all animal experiments (Charles River Discovery Services, MA, USA) is accredited by the Association for Assessment and Accreditation of Laboratory Animal Care International (AAALAC). To establish the systemic AML model, host bone marrow was ablated with cyclophosphamide, and NOD/SCID mice were thereafter intravenously inoculated with MV4-11 cells (Fig. S1A). After 3 weeks, we administered NTX-301 (1.5, 2.0, or 2.5 mg/kg), DAC (2.5 mg/kg), or AZA (5.0 mg/kg) according to the indicated schedules (Fig. S1A, B) and monitored alterations in the mouse survival and hematologic profiles until the end of the study (Day 87). Strikingly, compared with the intraperitoneal administration (i.p.) of AZA and DAC, oral administration (p.o.) of NTX-301 at all doses significantly prolonged overall survival (Fig. 1A); the median time to endpoint for NTX-301 was 73.5–85 days (Fig. 1B; 47 days for vehicle, 38.5 days for DAC, and 66 days for AZA). The increased life span (ILS) values were calculated (median time to endpoint in the treatment group)/(median time to endpoint in the control group)%–100%, and the ILS achieved by NTX-301 treatment (ILS_NTX-301_) was 60–88% (vs. ILS_AZA_ = 43%). DAC shortened the ILSs of all treated mice (ILS_DAC_ = −19%; Fig. 1B), indicating treatment-related toxicity. NTX-301-treated mice showed minimal to minor changes in blood counts and body weight, whereas AZA treatment resulted in larger changes in blood counts (e.g., a marked decrease in the neutrophil count on Day 39) (Fig. S2A, B). At the end of the experiment (moribundity or last day of the experiment), all available mice (*n* = 7) were euthanized to analyze residual MV4-11 cells. Flow cytometry analysis revealed that the proportion of human CD45^+^ MV4-11 cells accounted for 0–9.5% of living bone marrow cells isolated from NTX-301-treated mice (Fig. S3); four of seven mice had ≤0.17% residual AML cells.

**Fig. 1:**
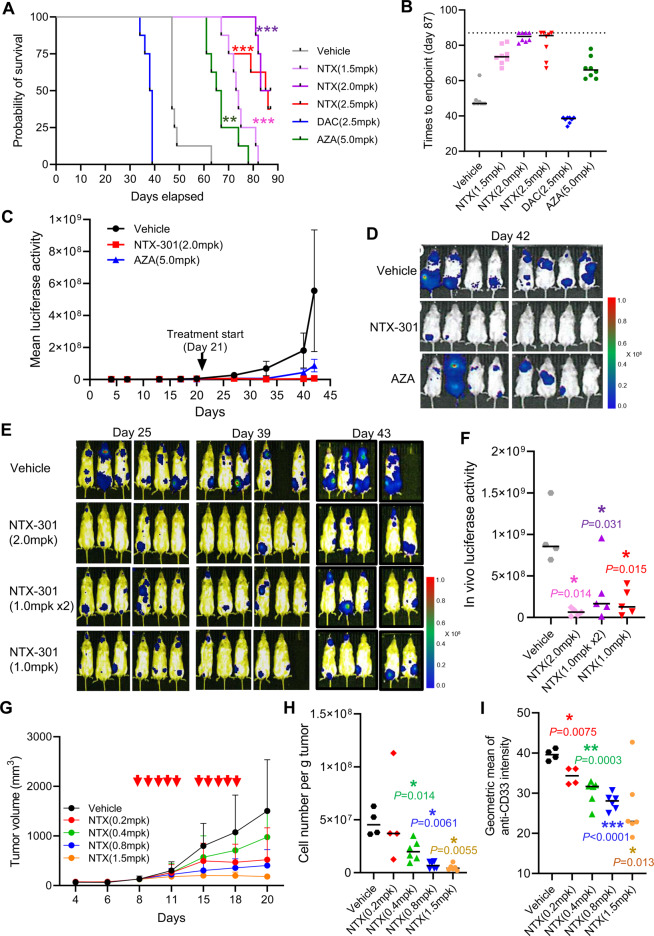
Preclinical models of AML demonstrated the improved therapeutic index of NTX-301. **A**, **B** A Kaplan–Meier plot of the survival probability (**A**) and a plot showing times to endpoint (moribund or the last day of study) (**B**) of female NOD/SCID mice bearing MV4-11 tumors (*n* = 8 per group, six groups) upon treatment with NTX-301 [(1.5, 2.0, or 2.5 mg/kg) (p.o.)], DAC [(2.5 mg/kg) (i.p.)], or AZA [5.0 mg/kg (i.p.)]. **C**, **D** Tumor growth measured by the quantification of bioluminescence emission (photons/sec) (**C**) and bioluminescence images on day 42 (**D**) in female NOD/SCID mice bearing MV4-11 tumors (*n* = 8 per group, three groups) upon treatment with NTX-301 [(2.0 mg/kg) (p.o.)] or AZA [(5.0 mg/kg) (i.p.)]. **E**, **F** Bioluminescence images (**E**) and quantification of bioluminescence emission (photons/sec) (**F**) of female NOD/SCID mice (*n* = 6 per group, four groups) bearing luciferase-labeled MV4-11 tumors upon treatment with NTX-301 [(daily at 1.0 or 2.0 mg/kg) or 2x daily at 1.0 mg/kg (p.o.)]. **G–I** Tumor growth (**G**), cell number per gram tumor (**H**), and anti-CD33 staining intensity (**I**) of female NMRI nude mice bearing subcutaneous MOLM-13 tumors (*n* = 6 per group, five groups) upon NTX-301 treatment [(0.2, 0.4, 0.8, or 1.5 mg/kg) (i.p.)]. In **G**, the red arrows denote the time points of NTX-301 treatment. AZA azacitidine, DAC decitabine, NTX NTX-301; mpk mg/kg, p.o. oral administration, i.p. intraperitoneal administration. *P*-values (vs. vehicle) are specified and marked as follows: *p < 0.05; **p < 0.001; ***p < 0.0001.

We then compared the antitumor efficacy of NTX-301 with that of AZA in a systemic NOD/SCID model bearing luciferase-labeled MV4-11 tumors (Fig. S1C); DAC, which showed treatment-related toxicity, was excluded. Bioluminescence imaging revealed that both agents were efficacious, but NTX-301 [2.0 mg/kg (p.o.)] eradicated tumors more effectively than AZA [5.0 mg/kg (i.p.)] (Fig. 1C, D; Fig. S4). In addition, using the same mouse model, NTX-301 treatment at different treatment doses and frequencies (Fig. S1D) resulted in marked tumor suppression under all administration conditions (Fig. 1E), with the highest efficacy being achieved when administered once daily at 2.0 mg/kg (Fig. 1F; 85% reduction vs. vehicle on Day 43). Finally, using a subcutaneous NMRI nude mouse model bearing MOLM-13 tumors (Fig. S1E), NTX-301 treatment [0.2–1.5 mg/kg (i.p.)] exhibited significant tumor suppression in a dose-dependent manner, notably resulting in tumor stasis at 1.5 mg/kg (Fig. 1G). Single-cell analysis using tumors harvested at the end of the experiment revealed that NTX-301 significantly decreased cell numbers normalized to tumor weights (Fig. 1H) and the population of human CD33^+^ AML cells in a dose-dependent manner (Fig. 1I). Collectively, these in vivo preclinical studies demonstrated the improved antileukemic activity, tolerability, and survival outcomes of NTX-301.

To elucidate the mechanisms of action (MoAs) underlying the improved antileukemic activity of NTX-301, we explored global transcriptome alterations in three AML cell lines (MV4-11, MOLM-13, and HL-60) upon treatment with NTX-301 or DAC. Consistent with its observed efficacy, NTX-301 promoted greater transcriptional reprogramming toward a normal myeloid-like signature [9] than DAC, accompanied by stronger suppression of the leukemic stem cell signature (Fig. S5A). Geneset enrichment analysis querying genes that were upregulated more strongly by NTX-301 than by DAC implied that the most significantly activated MoAs of NTX-301 were the DNA damage response (DDR) and the p53 pathway (Fig. S5B). Activation of the p53 pathway was observed specifically in p53-proficient MV4-11 and MOLM-13 cells but not in p53-null HL-60 cells (Fig. S5B). NTX-301 upregulated most of the 116 conserved p53 target genes [10], while DAC did so to a lesser extent (Fig. S6A). Consistently, the results of both Ingenuity pathway analysis (IPA) and geneset enrichment analysis (GSEA) indicated that NTX-301 triggered the p53 pathway more strongly than DAC (Fig. S6B, C).

To validate the activation of the DDR and the p53 pathway, we examined the phosphorylation of H2AX (a DDR marker) and CHK1 (a DDR sensor) and the stability of p53. Indeed, NTX-301 simultaneously stimulated more accumulation of pH2AX, pCHK1, and p53 than DAC (Fig. S7A). The accumulation of p53 may have been mediated by a posttranscriptional mechanism because NTX-301 did not affect the mRNA level of *TP53* but did upregulate the levels of p53 target genes, *CDKN1A* and *MDM2* (Fig. S7B).

Given the synthetic lethality of p53 activation and BCL-2 inhibition in AML [11, 12], NTX-301, with its ability to induce stronger p53 activation, may confer more benefits when used in combination with VCX than conventional HMAs. A recent genome-wide CRISPR/Cas9 screen systematically identified key components that drive sensitivity or resistance to VCX [12]. Application of these results to our transcriptome data revealed that NTX-301 up- and downregulated genes driving sensitivity and resistance, respectively, more strongly than DAC (Fig. 2A), thus priming AML cells for higher sensitivity to VCX. Indeed, when combined with VCX, both NTX-301 and DAC dramatically decreased the survival of AML cells in a dose- and p53-dependent manner (Fig. 2B). Importantly, the combination of NTX-301 + VCX exhibited higher efficacy (Fig. 2B) and resulted in a combination index (CI) implying greater synergy than DAC + VCX (Fig. 2C). Intriguingly, the CI of DAC + VCX was severely reduced by p53 loss-of-function, whereas the synergistic effect of NTX-301+VCX was sustained under these conditions (Fig. 2C; Fig. S7C).

**Fig. 2:**
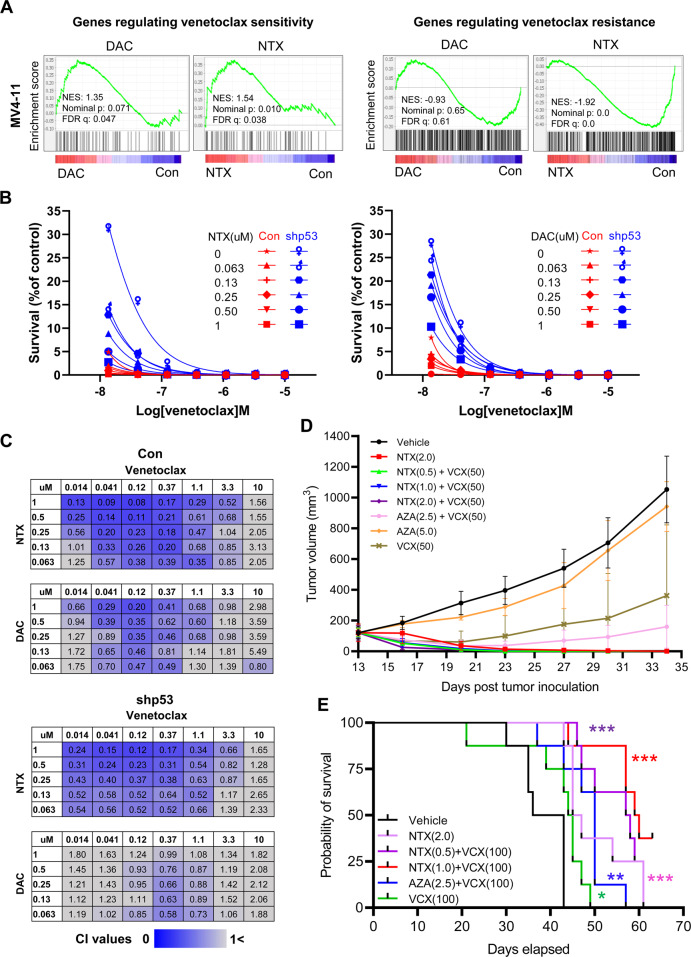
NTX-301 conferred benefits in combination with VCX. **A** GSEA plots showing significant enrichment of genes regulating sensitivity or resistance to VCX among transcriptome changes induced by 48 h of treatment with NTX-301 or DAC in MV4-11 cells. **B** Line plots showing the survival (%) of parental (Con) and *TP53*-knockdown (shp53) MV4-11 cells upon treatment with NTX-301 + VCX (left) or DAC + VCX (right) for 72 h. **C** Matrices showing the combination index (CI) values upon treatment with NTX-301+VCX or DAC + VCX for 72 h at the indicated concentrations in parental (top) and *TP53*-knockdown (bottom, shp53) MV4-11 cells. CI values < 1 (blue) indicate synergistic drug combination; darker blue colors are correlated with stronger the synergism, and CI values > 1 (gray) indicate no synergism. **D** Growth of MV4-11 tumors subcutaneously implanted into female BALB/c nude mice (*n* = 5 per group, eight groups) upon treatment with NTX-301 or AZA as a monotherapy or in combination with VCX. **E** Kaplan–Meier curves showing the survival probabilities of female NCG mice (*n* = 8 per group, six groups) intravenously injected with MV4-11 cells upon treatment with NTX-301 or AZA as a monotherapy or in combination with VCX. AZA azacitidine, DAC decitabine, NTX NTX-301, VCX venetoclax. *p < 0.05; **p < 0.01; ***p < 0.001.

We assessed the preclinical efficacy of combination therapy using both systemic and subcutaneous xenograft models (Fig. S8A, B). Strikingly, oral administration of NTX-301 + VCX achieved complete tumor remission (Fig. 2D), prolonged survival benefits (Fig. 2E), and a lack of notable weight loss in mouse models (Fig. S9A, B). Although the combination of AZA + VCX also effectively regressed tumors with no notable weight loss at early treatment stages, the tumors eventually rebounded (Fig. 2D). NTX-301 monotherapy was even superior to combination therapy with AZA + VCX in terms of efficacy and survival outcomes (Fig. 2D, E).

In conclusion, our findings highlight the improved therapeutic index of NTX-301 both as a monotherapy and in combination with VCX compared with those of conventional HMAs. Moreover, the oral administration of NTX-301 was superior to the intraperitoneal delivery of conventional HMAs, emphasizing its additional clinical advantage.

This study was limited to evaluating the efficacy of NTX-301 using primary AML samples derived from human patients. However, we expect to confirm the preliminary efficacy of NTX-301 in current ongoing clinical trials. Further mechanistic studies, especially in terms of the DDR and the p53-dependent MoA, are also required to understand why minor structural differences characterized as 4′-thio-modification confer stronger efficacy than conventional HMAs. Moreover, given that the combination of NTX-301+VCX maintained a stronger synergistic effect than DAC + VCX even under p53 depletion, the p53-independent MoA of NTX-301 should be investigated.

Our findings provide the rationale for the current ongoing clinical development of NTX-301 as a monotherapy (NCT04167917, NCT03366116, and NCT04851834). At the same time, our findings support a new launce of clinical trials on combination therapy with NTX-301+VCX. Therefore, we believe that NTX-301 can be a promising therapeutic option for elderly patients with AML and potentially serve as an alternative to the currently available HMAs.

## Supplementary information

Supplementary Figures & Legends

Supplementary Methods

Checklist file

## Data Availability

Data are available in NCBI GEO (https://www.ncbi.nlm.nih.gov/geo/) under accession numbers GSE188392 and GSE187285.

## References

[1] Kantarjian H, Kadia T, DiNardo C, Daver N, Borthakur G, Jabbour E (2021). Acute myeloid leukemia: current progress and future directions. Blood Cancer J.

[2] Short NJ, Rytting ME, Cortes JE (2018). Acute myeloid leukaemia. Lancet.

[3] Bell JA, Galaznik A, Huelin R, Stokes M, Guo Y, Fram RJ (2018). Effectiveness and safety of therapeutic regimens for elderly patients with acute myeloid leukemia: a systematic literature review. Clin Lymphoma Myeloma Leuk.

[4] Jonas BA, Pollyea DA (2019). How we use venetoclax with hypomethylating agents for the treatment of newly diagnosed patients with acute myeloid leukemia. Leukemia..

[5] Santini V, Ossenkoppele GJ (2019). Hypomethylating agents in the treatment of acute myeloid leukemia: a guide to optimal use. Crit Rev Oncol Hematol.

[6] DiNardo CD, Jonas BA, Pullarkat V, Thirman MJ, Garcia JS, Wei AH (2020). Azacitidine and venetoclax in previously untreated acute myeloid leukemia. N. Engl J Med.

[7] DiNardo CD, Pratz KW, Letai A, Jonas BA, Wei AH, Thirman M (2018). Safety and preliminary efficacy of venetoclax with decitabine or azacitidine in elderly patients with previously untreated acute myeloid leukaemia: a non-randomised, open-label, phase 1b study. Lancet Oncol.

[8] Pollyea DA, Pratz K, Letai A, Jonas BA, Wei AH, Pullarkat V (2021). Venetoclax with azacitidine or decitabine in patients with newly diagnosed acute myeloid leukemia: long term follow-up from a phase 1b study. Am J Hematol.

[9] van Galen P, Hovestadt V, Wadsworth Ii MH, Hughes TK, Griffin GK, Battaglia S (2019). Single-cell RNA-seq reveals AML hierarchies relevant to disease progression and immunity. Cell.

[10] Fischer M (2017). Census and evaluation of p53 target genes. Oncogene..

[11] Pan R, Ruvolo V, Mu H, Leverson JD, Nichols G, Reed JC (2017). Synthetic lethality of combined Bcl-2 inhibition and p53 activation in AML: mechanisms and superior antileukemic efficacy. Cancer Cell.

[12] Chen X, Glytsou C, Zhou H, Narang S, Reyna DE, Lopez A (2019). Targeting mitochondrial structure sensitizes acute myeloid leukemia to venetoclax treatment. Cancer Discov.

