# Supplementary material for: The preclinical efficacy of the novel hypomethylating agent NTX-301 as a monotherapy and in combination with venetoclax in acute myeloid leukemia
Source: Blood Cancer J. 2022 Apr 11;12(4):57. doi: 10.1038/s41408-022-00664-y (PMC9001641; doi:10.1038/s41408-022-00664-y)

# Supplementary Figure Legends

# The preclinical efficacy of the novel hypomethylating agent NTX-301 as a monotherapy and in combination with venetoclax in acute myeloid leukemia

**Running title: NTX-301 in acute myeloid leukemia**

Byungho Lim[1,*], Dabin Yoo[1], Younghwa Chun[2], Areum Go[2], Kyung-Jin Cho[1], Daeun Choi[1], Myoung Eun Jung[1], Ha Young Lee[2], Rebecca J. Boohaker[3], Jin Soo Lee[2], DooYoung Jung[2], and Gildon Choi[1,*]

[1]Research Center for Drug Discovery Technology, Korea Research Institute of Chemical Technology, Daejeon, Republic of Korea

[2]Pinotbio, Inc, Suwon, Republic of Korea

[3]Southern Research, Division of Drug Discovery, Birmingham, AL, United States

**Supplementary Figure S1. Summary of treatment schedules for assessing the efficacy and survival benefits of NTX-301 in four preclinical models of AML. A** Schematic outline of the 3-cycle NTX-301 treatment schedule for monitoring survival and hematologic profiles in the systemic MV4-11 model. **B-E** Tables summarizing the treatment schedules for monitoring survival and hematologic profiles in the systemic MV4-11 model (**B**), for comparing the antitumor efficacy of NTX-301 with that of AZA in the systemic model bearing luciferase-labeled MV4-11 tumors (**C**), for comparing the antitumor efficacy of NTX-301 administered by different methods in the systemic model bearing luciferase-labeled MV4-11 tumors (**D**) and for evaluating the antitumor efficacy of NTX-301 in a dose-dependent manner in the subcutaneous model bearing MOLM-13 tumors (**E**). CP, cyclophosphamide; AZA, azacitidine; DAC, decitabine; NTX, NTX-301; p.o., oral administration; i.p., intraperitoneal administration; q.d., once a day.

**Supplementary Figure S2. In vivo assessment of tolerability upon treatment with NTX-301, DAC, or AZA. A-B** Line plots showing changes in hematologic profiles (**A**) and body weights (**B**) of female NOD/SCID mice bearing MV4-11 tumors (n=8 per group) upon treatment with NTX-301 [(1.5, 2.0, or 2.5 mg/kg (p.o.)], DAC (2.5 mg/kg (i.p.)], or AZA [5.0 mg/kg (i.p.)]. AZA, azacitidine; DAC, decitabine; NTX, NTX-301; SEM, standard error of the mean; p.o., oral administration; i.p., intraperitoneal administration.

**Supplementary Figure S3. Flow cytometric analyses for the detection of**

**residual human CD45+ MV4-11 cells in cells isolated from bone marrow.** The inner boxes indicate human CD45+ MV4-11 cells, and the percentages of these cells are shown on the right.

**Supplementary Figure S4. Comparison of the in vivo efficacies of NTX-301 and AZA.** In NOD/SCID mouse models transplanted with luciferase-labeled MV4-11 cells (n=8 per group), tumor growth was measured by the quantification of bioluminescence emission (photons/sec) upon treatment with NTX-301 (2.0 mg/kg p.o.) or AZA (5.0 mg/kg i.p.). This Supplementary Figure presents single individual values from Fig. 1C to avoid misleading by large standard deviations. *P*-values (vs. vehicle) are specified and marked as follows: *, $p<0.05$; **, $p<0.001$; NS, not significant. AZA, azacitidine; p.o., oral administration; i.p., intraperitoneal administration.

**Supplementary Figure S5. Transcriptome-wide alterations induced by NTX-301 revealed MoAs of NTX-301. A** GSEA plots displaying significant enrichment of a normal myeloid-like signature (left) and a leukemic stem cell signature (right) among transcriptome changes induced by NTX-301 or DAC for 48 hours in MV4-11 and HL-60 cells. **B** Heatmaps showing genesets upregulated more strongly by NTX-301 than by DAC and bar plots showing the biological pathways associated with each gene set in MV4-11, MOLM-13, and HL-60 cells. DAC, decitabine; GSEA, gene set enrichment analysis; MoA, mechanism of action; NTX, NTX-301.

**Supplementary Figure S6. Transcriptome analyses implied that NTX-301 activates a p53 transcriptional program. A** Heatmap showing the expression levels of p53 target genes (n=116) induced by NTX-301 or DAC for 48 hours in MV4-11 cells. Genes with marked transcriptional changes are listed. **B** Ingenuity pathway analysis (IPA) highlighting the activation of p53 as an upstream regulator of genes upregulated by DAC or NTX-301. Z-scores and *p*-values are shown. **C** GSEA plots showing significant enrichment of a p53 signature among transcriptome changes induced by NTX-301 or DAC for 48 hours in p53-proficient AML cells, MV4-11 and MOLM-13. DAC, decitabine; GSEA, gene set enrichment analysis; NTX, NTX-301.

**Supplementary Figure S7. NTX-301 elicited the induction of the DDR and the p53 pathway. A** Western blots showing the expression levels of proteins involved in the DDR-p53 pathway in MV4-11 cells treated with NTX-301 or DAC (60, 200, and 500 nM) for 24 hours (up). Western blot showing the protein levels of DNMT1 in MV4-11, MOLM-13, and HL-60 cells treated with NTX-301 or DAC (60, 200, and 500 nM) for 24 hours (bottom). **B** Bar plots showing the mRNA expression levels ($log_2$) of *TP53*, *MDM2*, and *CDKN1A* upon treatment with NTX-301 or DAC (60 nM) for 48 and 96 hours in the MV4-11 cell line. **C** Western blots showing the expression levels of p53 in parental (Con) and *TP53*-knockdown (shTP53) MV4-11 cells. DAC, decitabine; DDR, DNA damage response; NTX, NTX-301.

**Supplementary Figure S8. Summary of the treatment schedules for assessing the efficacy of NTX-301 as a monotherapy and in combination with VCX using**

**two preclinical models of AML. A-B** Tables summarizing the treatment schedules for comparing the combinatorial efficacies of NTX-301+VCX and AZA+VCX in the subcutaneous MV4-11 model (**A**) and for comparing the survival benefits of NTX-301+VCX and AZA+VCX in the systemic MV4-11 model (**B**). AZA, azacitidine; NTX, NTX-301; VCX, venetoclax; p.o., oral administration; i.p., intraperitoneal administration; q.d., once a day; biw, twice weekly.

**Supplementary Figure S9. Assessment of mouse body weights upon treatment with NTX-301 or AZA as a monotherapy and in combination with VCX using two preclinical models of AML. A-B** Line plots showing changes in the body weights of the mice described in Supplementary Fig. S8A (**A**) and Supplementary Fig. S8B (**B**). AZA, azacitidine; NTX, NTX-301; VCX, venetoclax.

## Supplementary Figure S1

**A**

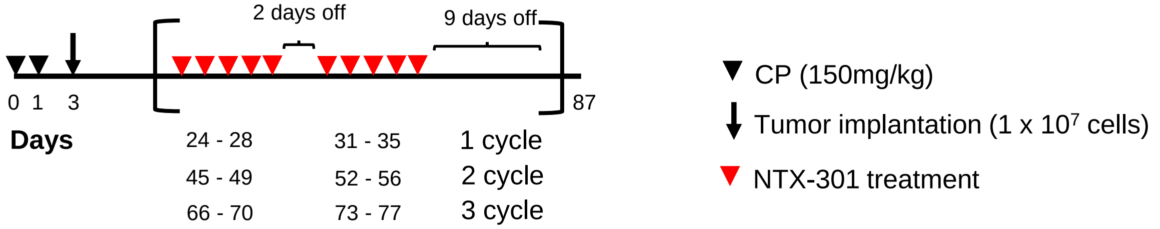


**B**

| Group | Mice | Agent | mg/kg | Route | Schedule |
|---|---|---|---|---|---|
| 1 | n=8 | Vehicle | --- | po | qd x 5 then 2 days off, 5 days on, 9 days off for 3 cycles |
| 2 | n=8 | NTX-301 | 1.5 | po | qd x 5 then 2 days off, 5 days on, 9 days off for 3 cycles |
| 3 | n=8 | NTX-301 | 2.0 | po | qd x 5 then 2 days off, 5 days on, 9 days off for 3 cycles |
| 4 | n=8 | NTX-301 | 2.5 | po | qd x 5 then 2 days off, 5 days on, 9 days off for 3 cycles |
| 5 | n=8 | DAC | 2.5 | ip | Three times a week for two weeks |
| 6 | n=8 | AZA | 5.0 | ip | Twice a week for three weeks |

**C**

| Group | Mice | Agent | mg/kg | Route | Schedule |
|---|---|---|---|---|---|
| 1 | n=8 | Vehicle | --- | po | Daily on Days 21-25 and 28-32 |
| 2 | n=8 | NTX-301 | 2.0 | po | Daily on Days 21-25 and 28-32 |
| 3 | n=8 | AZA | 5.0 | ip | Twice a week on days 21, 25, 28, 32, 35, and 39 |

**D**

| Group | Mice | Agent | Single dose (mg/kg) | Daily dose (mg/kg) | Route | Schedule |
|---|---|---|---|---|---|---|
| 1 | n=6 | Vehicle | --- | --- | po | Daily on Days 18-22, 25-29, and 39-43 |
| 2 | n=6 | NTX-301 | 2.0 | 2.0 | po | Daily on Days 18-22, 25-29, 39-43, and 46-48 |
| 3 | n=6 | NTX-301 | 1.0 | 2.0 | po | 2X daily on Days 18-22, 25-29, 39-43, and 46-47 and once on Day 48 |
| 4 | n=6 | NTX-301 | 1.0 | 1.0 | po | Daily on Days 18-22, 25-29, 32-36, 39-43, and 46-489 |

**E**

| Group | Mice | Agent | mg/kg | Route | Schedule |
|---|---|---|---|---|---|
| 1 | n=6 | Vehicle | --- | ip | 1x daily on days 8-12 and 15-19 |
| 2 | n=6 | NTX-301 | 0.2 | ip | 1x daily on days 8-12 and 15-19 |
| 3 | n=6 | NTX-301 | 0.4 | ip | 1x daily on days 8-12 and 15-19 |
| 4 | n=6 | NTX-301 | 0.8 | ip | 1x daily on days 8-12 and 15-19 |
| 5 | n=6 | NTX-301 | 1.5 | ip | 1x daily on days 8-12 and 15-19 |

## Supplementary Figure S2

**A**

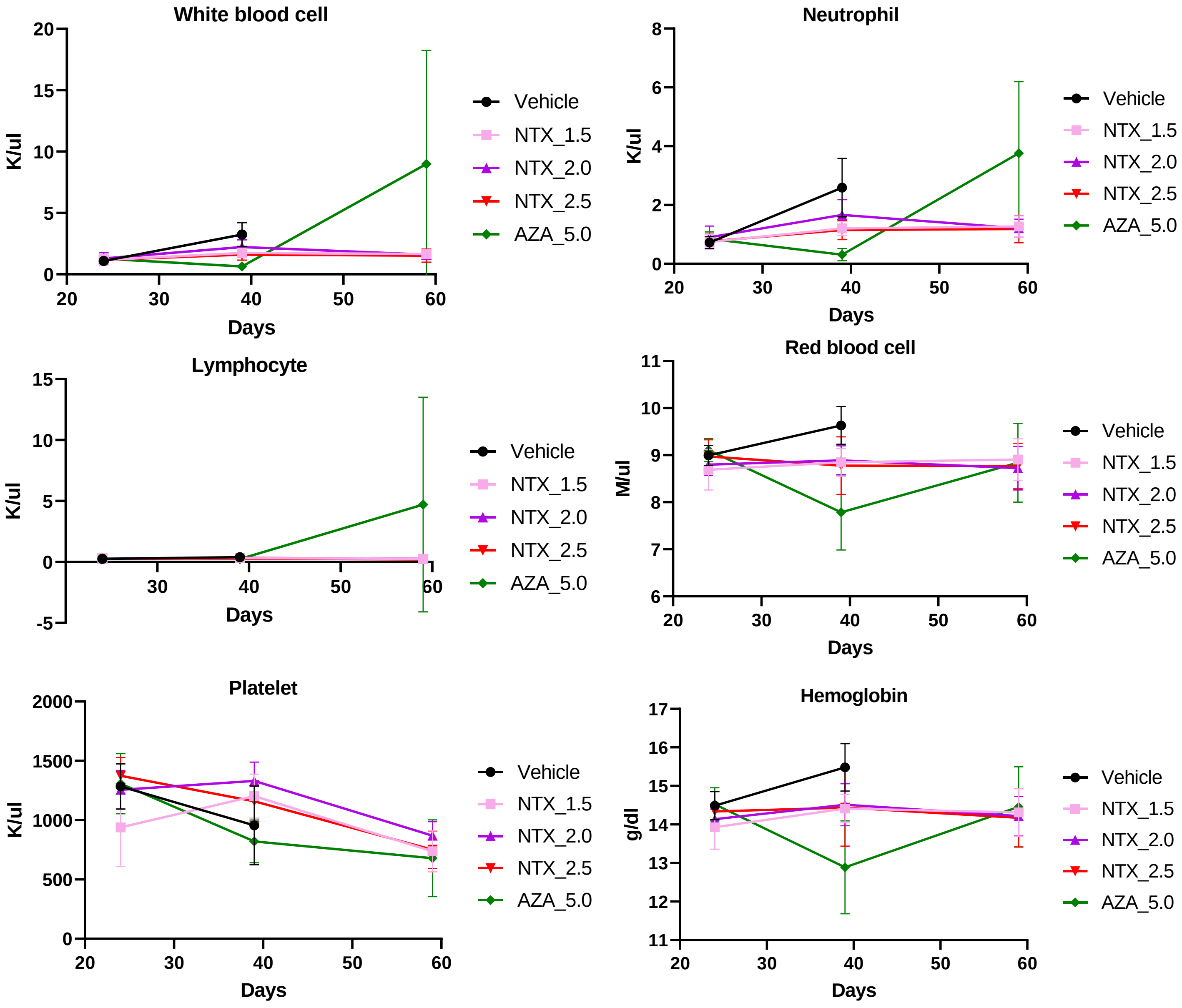


**B**

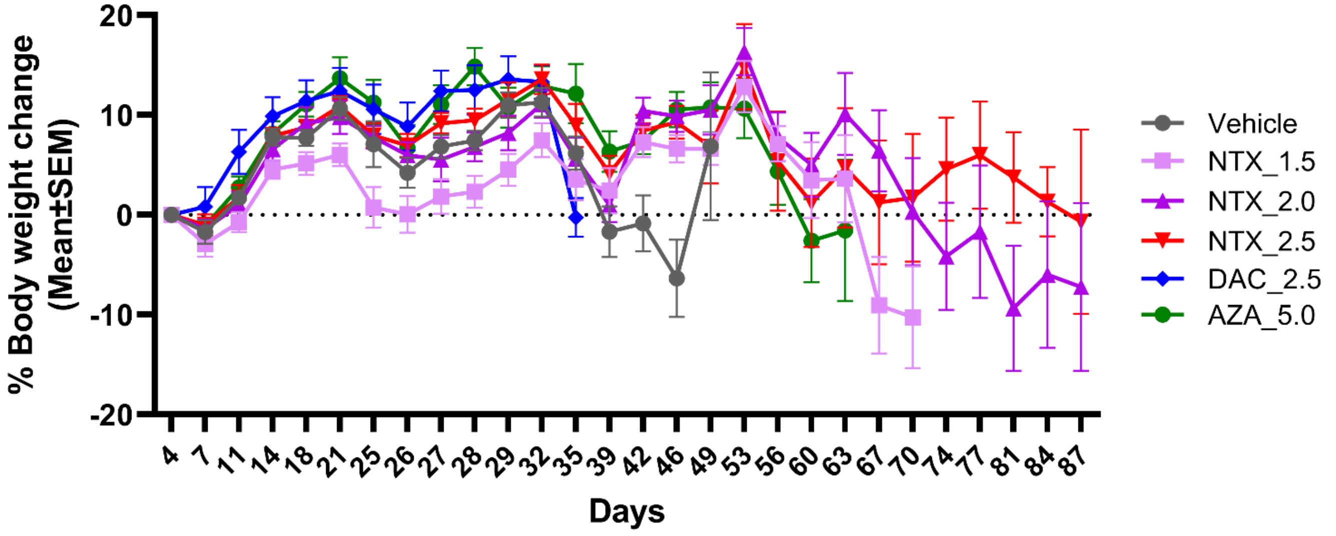

# Supplementary Figure S3

## % Human CD45+ MV4-11 cells determined by FACS analysis

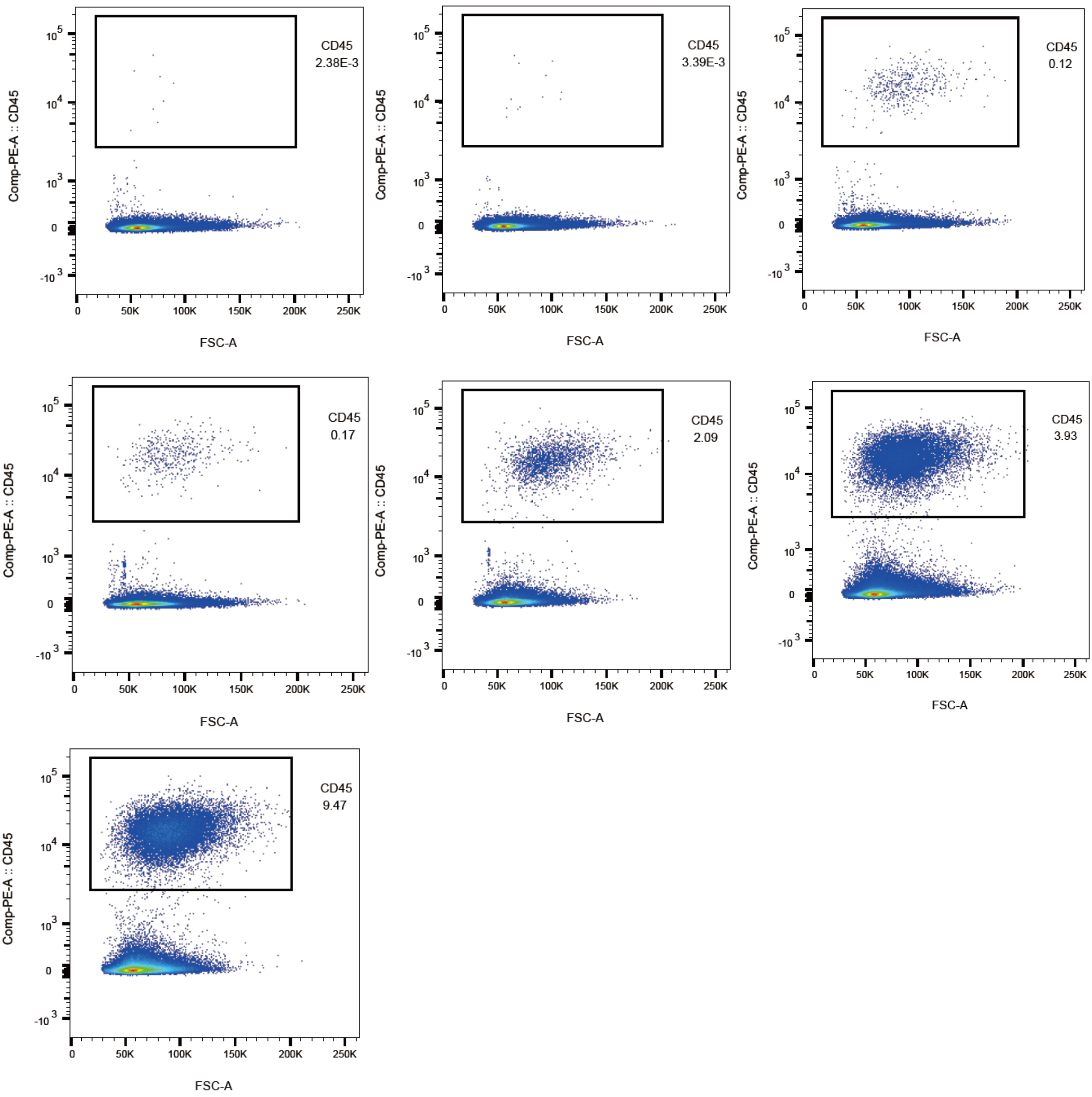

# Supplementary Figure S4

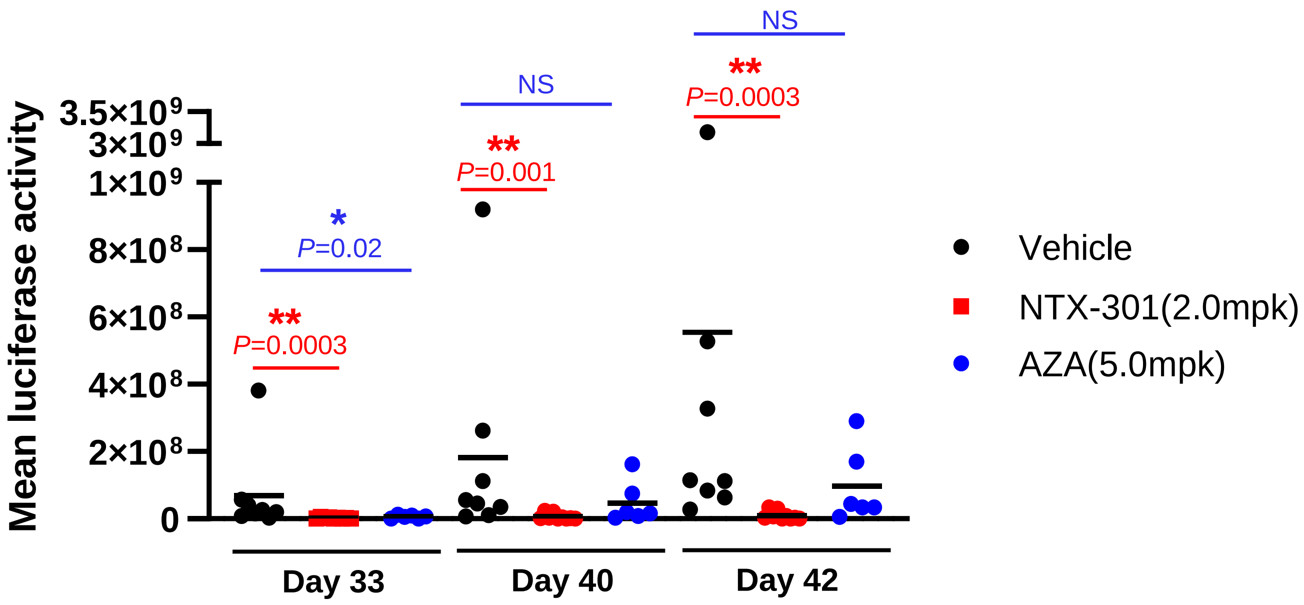

# Supplementary Figure S5

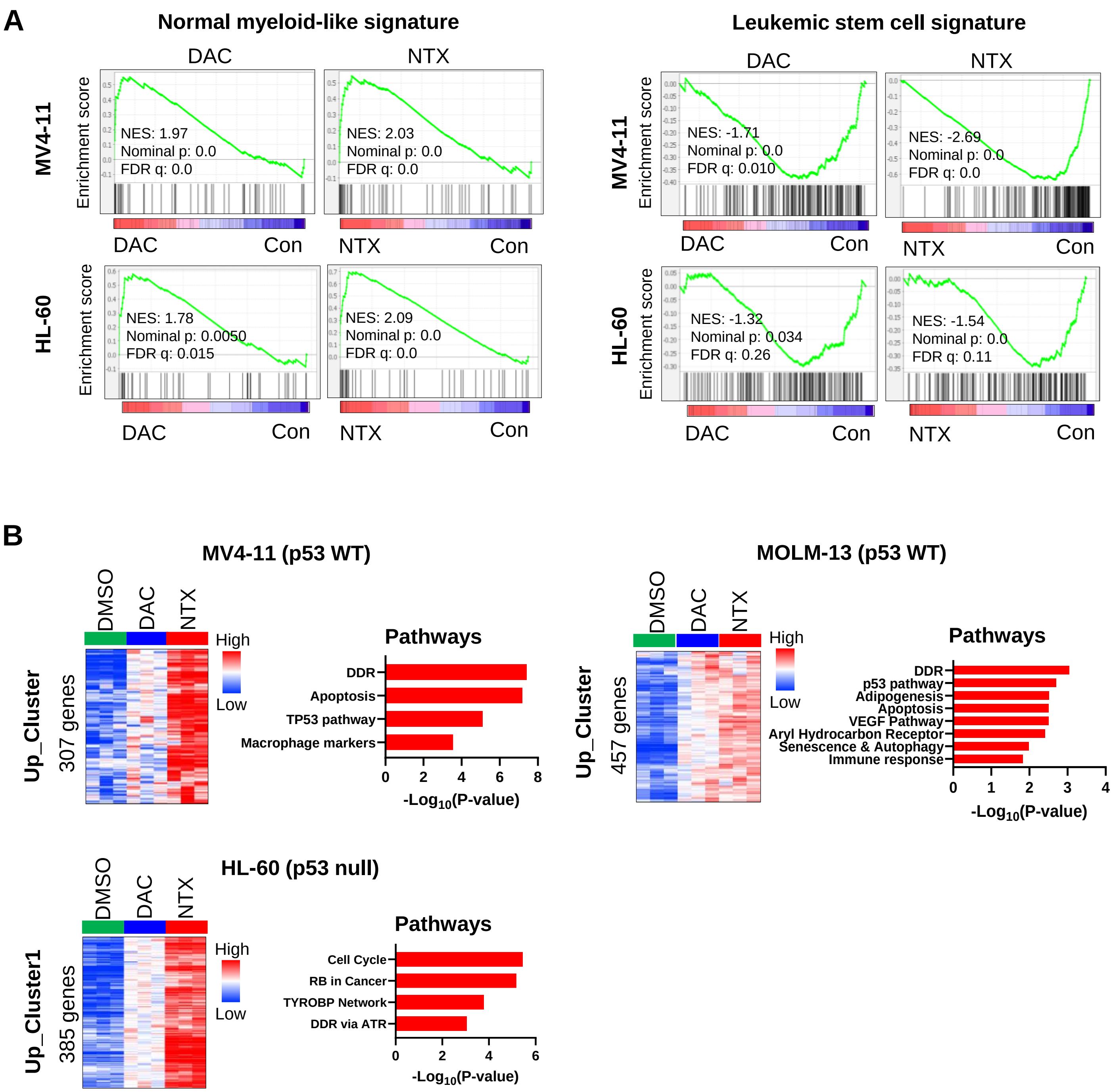

# Supplementary Figure S6

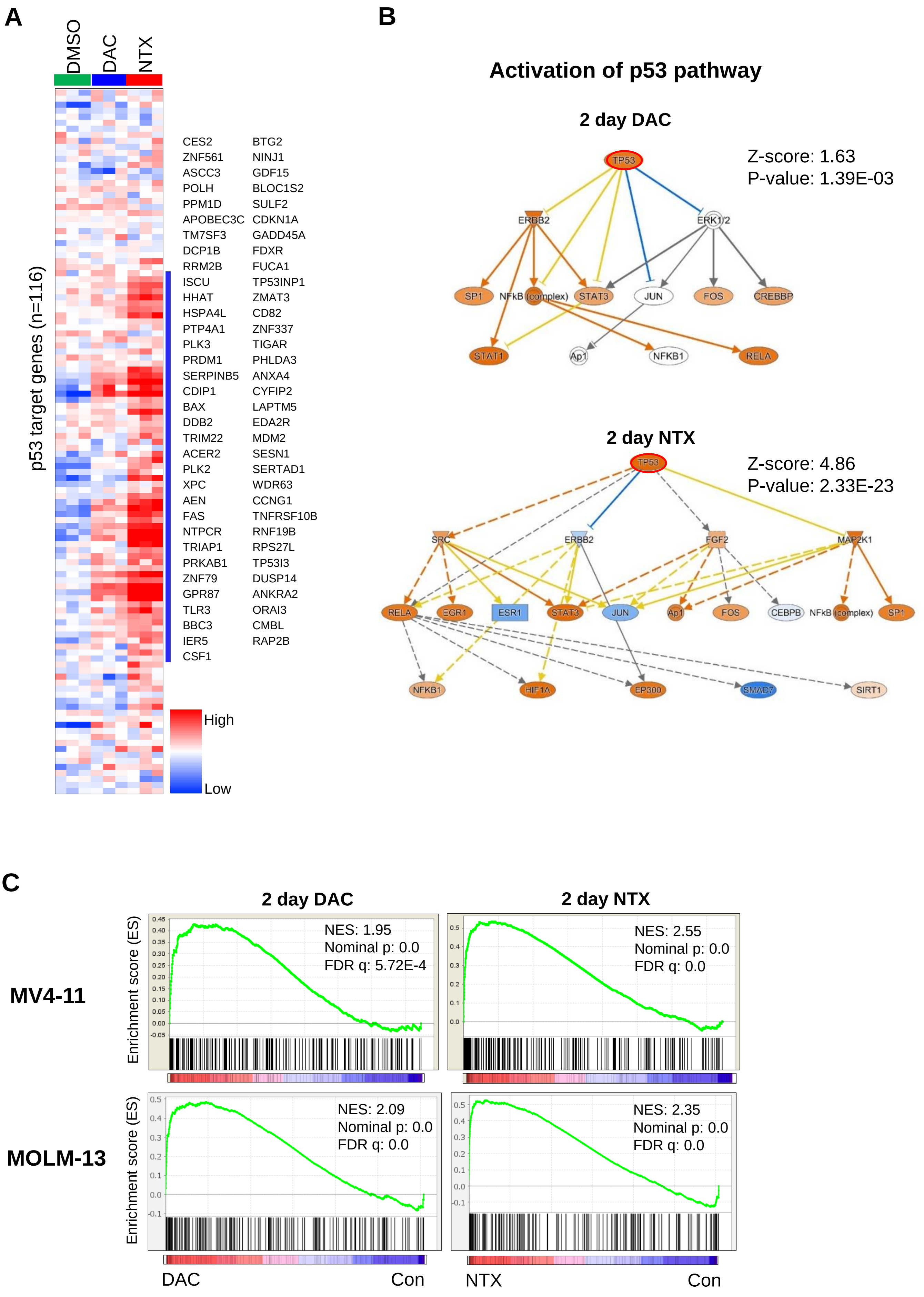

## Supplementary Figure S7

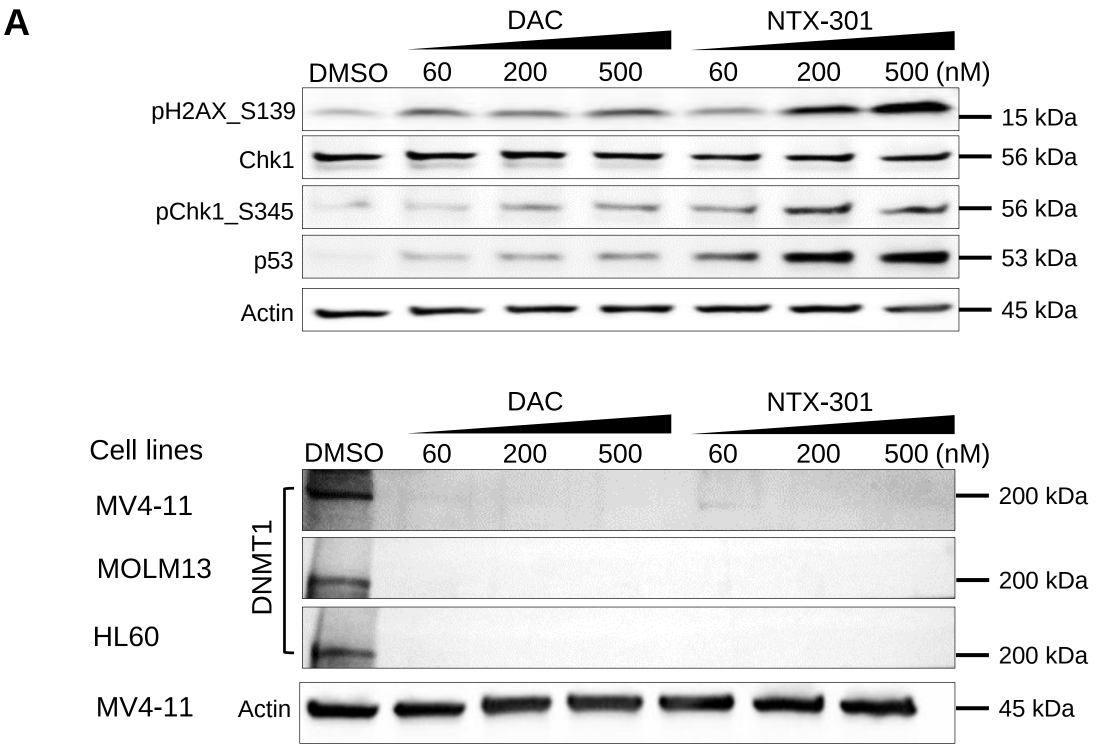


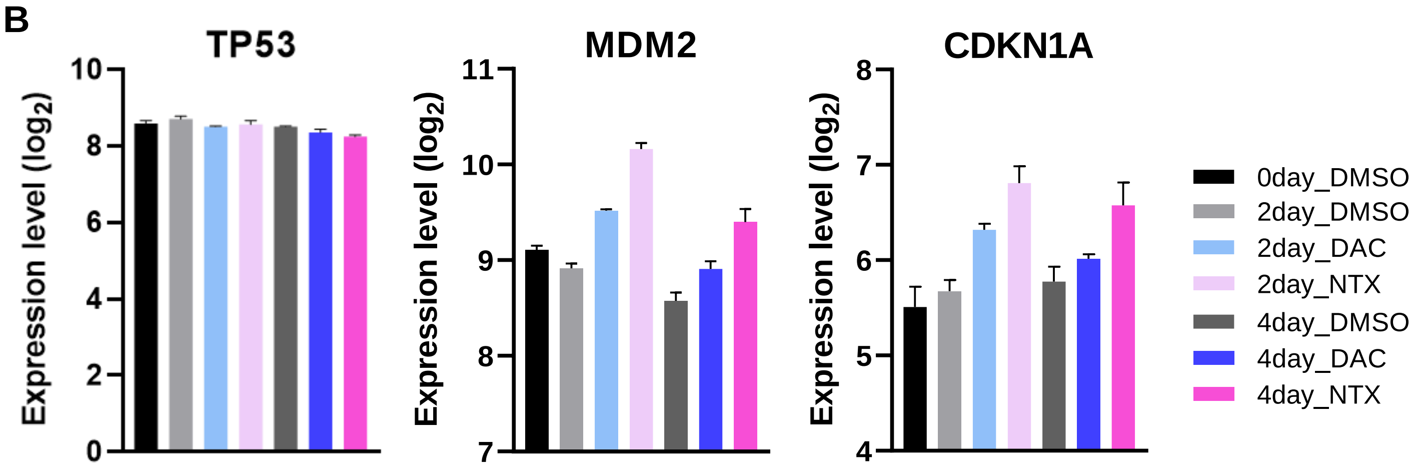


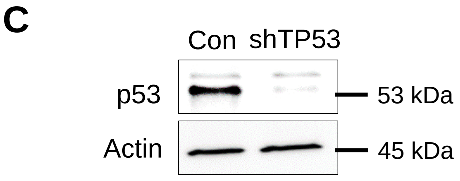

## Supplementary Figure S8

### A

| Group | Mice | Drug | Dose (mg/kg) | Route | Regimen |
|---|---|---|---|---|---|
| 1 | 5 | Vehicle | - | po | qd×5, 2 off, qd×5, 9 off for 3 cycles |
| 2 | 5 | NTX-301 | 2 | po | qd×5, 2 off, qd×5, 9 off for 3 cycles |
| 3 | 5 | NTX+VCX | 0.5 + 50 | po<br>po | qd×5, 2 off, qd×5, 9 off for 3 cycles<br>(qd×5, 2 off) ×3wks |
| 4 | 5 | NTX+VCX | 1 + 50 | po<br>po | qd×5, 2 off, qd×5, 9 off for 3 cycles<br>(qd×5, 2 off) ×3wks |
| 5 | 5 | NTX+VCX | 2 + 50 | po<br>po | qd×5, 2 off, qd×5, 9 off for 3 cycles<br>(qd×5, 2 off) ×3wks |
| 6 | 5 | AZA+VCX | 2.5 + 50 | ip<br>po | biw×3wks<br>(qd×5, 2 off) ×3wks |
| 7 | 5 | Azacitidine | 5 | ip | biw×3wks |
| 8 | 5 | Venetoclax | 50 | po | (qd×5, 2 off) ×3wks |

### B

| Group | Mice | Drug | Dose (mg/kg) | Route | Regimen |
|---|---|---|---|---|---|
| 1 | 8 | Vehicle | - | po | qd×5, 2 off, qd×5, 9 off for 3 cycles |
| 2 | 8 | NTX-301 | 2 | po | qd×5, 2 off, qd×5, 9 off for 3 cycles |
| 3 | 8 | NTX+VCX | 0.5 + 100 | po<br>po | qd×5, 2 off, qd×5, 9 off for 3 cycles<br>(qd×5, 2 off) × 9 |
| 4 | 8 | NTX+VCX | 1 + 100 | po<br>po | qd×5, 2 off, qd×5, 9 off for 3 cycles<br>(qd×5, 2 off) × 9 |
| 5 | 8 | AZA+VCX | 2.5 + 100 | ip<br>po | Biw × 9<br>(qd×5, 2 off) × 9 |
| 6 | 8 | VCX | 100 | po | (qd×5, 2 off) × 9 |

Supplementary Figure S9

A

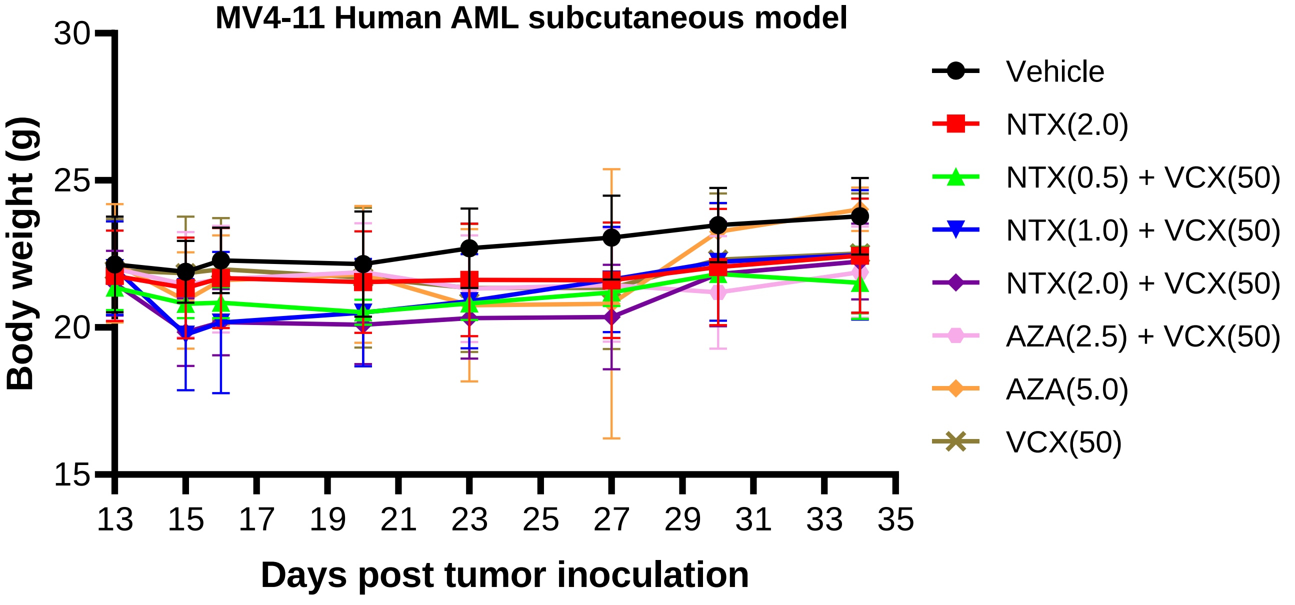


B

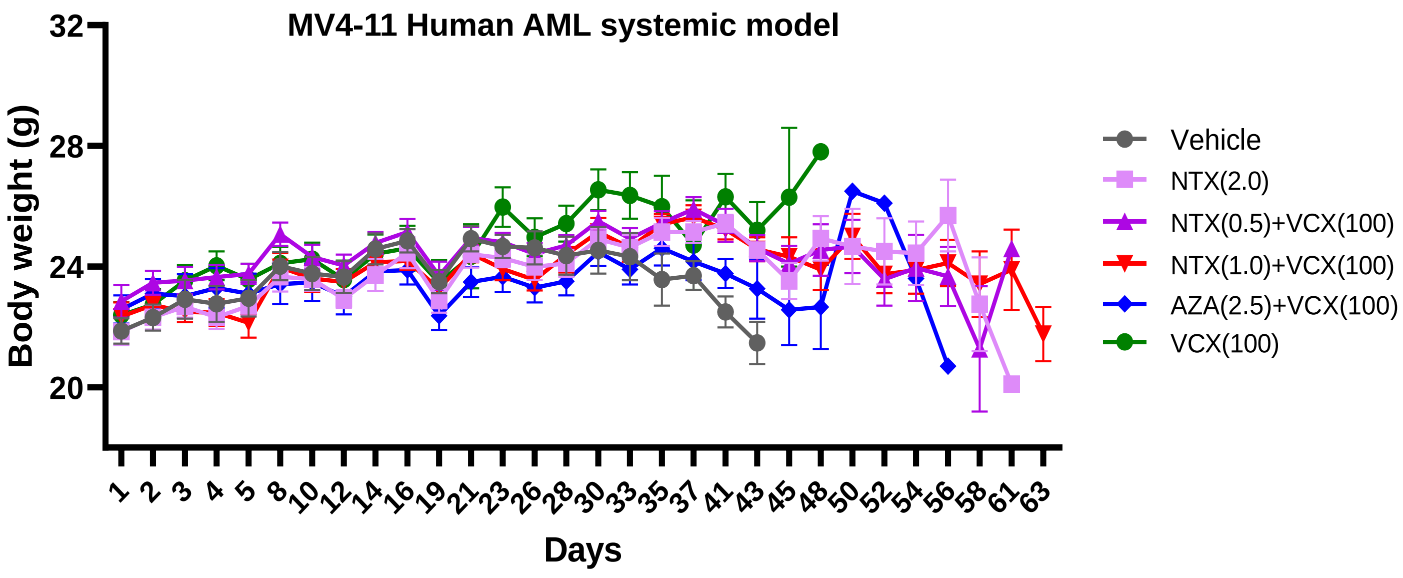

Supplement: Supplementary file 1 — Supplementary Figures & Legends [file 41408_2022_664_MOESM1_ESM.pdf]
