# Supplementary material for: The preclinical efficacy of the novel hypomethylating agent NTX-301 as a monotherapy and in combination with venetoclax in acute myeloid leukemia
Source: Blood Cancer J. 2022 Apr 11;12(4):57. doi: 10.1038/s41408-022-00664-y (PMC9001641; doi:10.1038/s41408-022-00664-y)
Supplement: Supplementary file 2 — Supplementary Methods [file 41408_2022_664_MOESM2_ESM.docx]

**Supplementary Methods**

**The preclinical efficacy of the novel hypomethylating agent NTX-301 as a monotherapy and in combination with venetoclax in acute myeloid leukemia**

**Running title: NTX-301 in acute myeloid leukemia**

Byungho Lim^1,*^, Dabin Yoo^1^, Younghwa Chun^2^, Areum Go^2^, Kyung-Jin Cho^1^, Daeun Choi^1^, Myoung Eun Jung^1^, Ha Young Lee^2^, Rebecca J. Boohaker^3^, Jin Soo Lee^2^, DooYoung Jung^2^, and Gildon Choi^1,*^

^1^Research Center for Drug Discovery Technology, Korea Research Institute of Chemical Technology, Daejeon, Republic of Korea

^2^Pinotbio, Inc, Suwon, Republic of Korea

^3^Southern Research, Division of Drug Discovery, Birmingham, AL, United States

**Cell lines and reagents**

HEK293T (ATCC, CRL-3216) cells and three human leukemia cell lines [MV4-11 (ATCC, CRL-9591), HL-60 (ATCC, CCL-240), and MOLM-13 (DSMZ, ACC 554)] were cultured in DMEM and RPMI, respectively. None of the cell lines were recently authenticated and tested for mycoplasma contamination, but no indication of contamination was observed. To establish stable cells, MISSION® TRC shRNA and Lentiviral Packaging Mix (Sigma, MA, USA) were cotransfected into 293T cells. Lentivirus-containing supernatants were collected over a two-day period and used for the transduction of MV4-11 cells, followed by puromycin selection (Thermo, MA, USA). NTX-301 was purchased from MercachemSyncom (Netherlands) and formulated in N-methyl-2-pyrrolidone (NMP) and then PEG400 followed by the addition of saline with vortexing and sonication for animal studies. DAC, AZA, and VCX were purchased from Selleckchem (TX, USA).

**Mouse studies**

All animal experiments were performed in compliance with the recommendations of the Guide for Care and Use of Laboratory Animals. The animal care and use program at Charles River Discovery Services (CR Discovery Services, MA, USA) is accredited by the Association for Assessment and Accreditation of Laboratory Animal Care International (AAALAC). This study includes six mouse models as described below (also summarized in Fig. S1 and Fig. S8). In all mouse models, n≥5 was chosen as the minimal sample size (n=8 for survival analyses), but no statistical methods for sample size estimation were used. To allocate mice into experimental groups, the mice were randomized and sorted by group mean body weight, but no blinding was performed.

1) Systemic female NOD/SCID mouse model (n=8 per group, 6 groups) to assess survival and complete blood count (CBC): To ablate host bone marrow, animals were pretreated with cyclophosphamide [(150 mg/kg (i.p.) once a day for two days] three days prior to tumor implantation. On the day of tumor implantation (Day 1), 1.0 X 10^7^ MV4-11 cells were intravenously inoculated via tail vein injection. After three weeks, the mice were randomized and sorted by body weight, ranging from 20.5 to 21.0 grams. Treatment was initiated according to Fig. S1B. Whole blood samples collected with the anticoagulant K2EDTA were shipped on wet ice to Antech (CA, USA) for CBC profiling.

2) Systemic female NOD/SCID models (n=8 per group, 3 groups) to compare the efficacy of NTX-301 and AZA: To ablate host bone marrow, animals were pretreated with cyclophosphamide [(100 mg/kg (i.p.) once a day for two days] three days prior to tumor implantation, and 5.0 X 10^6^ luciferase-labeled MV4-11 cells were injected intravenously. After randomization on Day 20, treatment was initiated according to Fig. S1C, and tumor growth was monitored using in vivo bioluminescence imaging.

3) Systemic female NOD/SCID models (n=6 per group, 4 groups) to assess the efficacy of NTX-301 at different treatment doses and frequencies: To ablate host bone marrow, animals were pretreated with cyclophosphamide [(100 mg/kg (i.p.) once a day for two days] three days prior to tumor implantation, and 5.0 X 10^6^ luciferase-labeled MV4-11 cells were injected intravenously. After randomization on Day 18, treatment was initiated according to Fig. S1D, and tumor growth was monitored using in vivo bioluminescence imaging.

4) Subcutaneous female NMRI nude mouse models (n=6 per group, 5 groups) to evaluate the efficacy of NTX-301 in a dose-dependent manner: 5.0 X 10^6^ MOLM-13 cells were subcutaneously injected into the left flank of each mouse (Day 0). After randomization by the caliper measurement of tumors (Day 8), treatment was initiated on the same day according to Fig. S1E. Tumor growth was monitored by manual calipering using the formula A X B^2^/2, where “A” and “B” are the long and short diameters of a tumor, respectively. Tumor tissues collected by necropsy (Day 20) were used to determine the number of single cells per gram tumor and to define human CD33^+^ populations using flow cytometry.

5) Subcutaneous female BALB/c nude mice (n=5 per group, 8 groups) to evaluate the efficacy of NTX-301 alone and in combination with VCX: 1.0 X 10^7^ MV4-11 cells were subcutaneously injected. Mice were randomized, and dosing was initiated according to Fig. S8A. Tumor growth was measured with a caliper.

6) Systemic female NCG mice (Prkdc^em26Cd52^Il2rg^em26Cd22^/NjuCrl, n=8 per group, 6 groups) to evaluate the survival outcomes of NTX-301 alone and in combination with VCX: 1.0 X 10^7^ MV4-11 cells were intravenously injected into the tail vein. Two weeks after implantation, mice were randomized, and dosing was initiated according to Fig. S8B.

**Western blotting**

Cells were lysed with NP-40 buffer (Thermo) and 30 µg of protein was loaded onto Mini-PROTEAN precast gels (Bio-Rad, CA, USA). Transfer was conducted using a Trans-Blot Turbo Transfer System (Bio-Rad). Immunoreactions were detected with SuperSignal™ West Pico or Femto substrates (Thermo) using an iBright System (Thermo). The following antibodies were used in this study and were purchased from Cell Signaling Technology: DNMT1 (#5032), pH2AX (#9718), p53 (#9282), Chk1 (#2360), pChk1 (#2348), and Actin (#4967).

**Data analysis**

Macrogen (Korea) produced the transcriptome data using RNA sequencing and the Affymetrix GeneChip® Human Gene 2.0 ST Array (CA, USA). Sequencing libraries were prepared using a TruSeq Stranded Total RNA LT Sample Prep Kit, and paired-end sequencing was conducted on the Illumina platform. Sequencing reads were processed using FastQC for quality control, Trimmomatic 0.38 for trimming [1], HISAT2 for mapping [2], and StringTie for transcript assembly and quantification [3]. Enrichr [4], GSEA [5, 6], IPA (QIAGEN, Netherlands), and Gene Ontology analyses were performed to query geneset-associated biological processes.

**Statistical analysis**

GraphPad Prism v9.0 (GraphPad Software) was used for statistical and graphical analyses. To confirm that all data followed a normal distribution, the Shapiro-Wilk normality test was used. In scatter plots, the two-tailed unpaired t-test was used to compare the mean differences between two groups when variances of two groups were similar as determined by the F-test, whereas the two-tailed unpaired t-test with Welch’s correction was applied when variances of two groups were different. The log-rank (Mantel-Cox) test was employed to determine the significance of the difference between the overall survival of the two groups in the Kaplan-Meier curves. Experiments were performed in at least triplicate, and the results are presented as follows: the group means ± standard errors of the mean (SEM) for Fig. 1C, Fig. 2B, and 2D, the group means ± 95% confidence intervals (CI) for Fig. 1G, and median-centered scatter plots for Fig. 1B, 1F, 1H, and 1I. Outliers identified from animal studies were not excluded from the statistical analysis. The combination index (CI) was calculated with CompuSyn. Statistical significance was defined as *p*<0.05.

**Data Availability**

Data are available in the NCBI GEO (https://www.ncbi.nlm.nih.gov/geo/) under accession numbers GSE188392 and GSE187293.

**References**

1. Bolger AM, Lohse M, Usadel B. Trimmomatic: a flexible trimmer for Illumina sequence data. Bioinformatics. 2014;30:2114-20.

2. Kim D, Langmead B, Salzberg SL. HISAT: a fast spliced aligner with low memory requirements. Nat Methods. 2015;12:357-60.

3. Pertea M, Pertea GM, Antonescu CM, Chang TC, Mendell JT, Salzberg SL. StringTie enables improved reconstruction of a transcriptome from RNA-seq reads. Nat Biotechnol. 2015;33:290-5.

4. Chen EY, Tan CM, Kou Y, Duan Q, Wang Z, Meirelles GV, et al. Enrichr: interactive and collaborative HTML5 gene list enrichment analysis tool. BMC Bioinformatics. 2013;14:128.

5. Mootha VK, Lindgren CM, Eriksson KF, Subramanian A, Sihag S, Lehar J, et al. PGC-1alpha-responsive genes involved in oxidative phosphorylation are coordinately downregulated in human diabetes. Nat Genet. 2003;34:267-73.

6. Subramanian A, Tamayo P, Mootha VK, Mukherjee S, Ebert BL, Gillette MA, et al. Gene set enrichment analysis: a knowledge-based approach for interpreting genome-wide expression profiles. Proc Natl Acad Sci U S A. 2005;102:15545-50.
